# ‘Follow the science’: Popular trust in scientific experts during the coronavirus pandemic

**DOI:** 10.1177/09636625241253968

**Published:** 2024-06-12

**Authors:** Ben Seyd, Joseph A Hamm, Will Jennings, Lawrence McKay, Viktor Valgarðsson, Meridith Anness

**Affiliations:** University of Kent, UK; Michigan State University, USA; University of Southampton, UK; Michigan State University, USA

**Keywords:** coronavirus, risk communication, science attitudes and perceptions, science experts, scientists attitudes, trust

## Abstract

The coronavirus pandemic increased the role played by scientific advisers in counselling governments and citizens on issues around public health. This raises questions about how citizens evaluate scientists, and in particular the grounds on which they trust them. Previous studies have identified various factors associated with trust in scientists, although few have systematically explored a range of judgements and their relative effects. This study takes advantage of scientific advisers’ heightened public profile during the pandemic to explore how people’s trust in scientists is shaped by perceptions of their features and traits, along with evaluations of their behaviour and role within the decision-making process. The study also considers people’s trust in politicians, thereby enabling us to identify whether trust in scientists reflects similar or distinctive considerations to trust in partisan actors. Data are derived from specially designed conjoint experiments and surveys of nationally representative samples in Britain and the United States.

The coronavirus pandemic underscored the central role played by scientists in guiding and informing governments and citizens on issues around public health and risk. In many countries, scientists were often key public communicators and figureheads of national responses to the pandemic. Most citizens, however, lacked the detailed technical knowledge of the coronavirus and its associated disease, COVID-19, to independently evaluate scientific experts and their guidance. Instead, many are likely to have fallen back on heuristic evaluations, notably judgements about whether those scientists could be trusted (Hovland et al., 1953; Pornpitakpan, 2004; Siegrist and Cvetkovich, 2000). In turn, trust in key information sources is vital if the public is to be encouraged to comply with collectively beneficial rules and constraints around social risk (Devine et al., 2024; Seyd and Bu, 2022). Given the importance of trust as a resource, it is imperative that we understand more clearly why citizens trust scientists as sources of information and expert guidance.

The bases of popular trust in scientists have been explored in numerous studies, which have identified a range of relevant factors or considerations (e.g. Besley et al., 2021). Most studies, however, focus on a limited number of factors and therefore tell us little about how far individuals’ trust in scientists might rest on multiple considerations, or about the relative effects of different considerations. Yet, scientists’ nature and work cannot be reduced to one or two features; instead, individuals are likely to judge scientists across a number of characteristics and behaviours, and appraisals of trust may be more or less linked to any of them. In addition, people’s trust in scientists may be shaped not only by perceptions of scientists’ traits and of the way they work – which have been the focus of most extant studies – but also by evaluations of their place in the decision-making process. Scientists’ prominence during the coronavirus pandemic intensified public debates about their role within the policy process, and about the appropriate balance in policy decisions between scientific evidence and wider economic and social values (Koch and Durodié, 2022). These considerations may well have played a role in shaping individuals’ judgements about scientists’ trustworthiness.

This study explores how people’s trust in scientists is shaped by judgements about different features of these actors’ traits, activities and work. Using an original survey, including a conjoint experiment, fielded in two countries – Britain and the United States – we explore the associations with trust of a range of evaluations, covering scientists’ competence, honesty, transparency, representativeness, communication style, relationship with politicians and consideration of wider social values. Our results confirm previous analyses which identify competence as a central consideration shaping people’s trust in scientists. Yet, our results also highlight other important considerations for trust, such as how scientists are seen to relate to the wider population, their behaviour, and the status of their guidance and advice to policy-makers. The study therefore extends previous analyses by highlighting the multiplicity of factors shaping public trust in scientists, and by showing how these factors extend beyond scientists’ general traits to encompass their behaviour and position within the decision-making process. The study also probes the role these factors play in shaping people’s trust in politicians, thereby enabling us to compare the bases of people’s trust in scientific experts and partisan political actors.

## 1. Public trust in scientists

We follow the majority of studies in science communication and risk analysis by treating trust as an evaluation or appraisal; a summative or latent belief that an actor or information source manifests qualities that render them trustworthy.^1^ While these trustworthy qualities are often defined in broad terms, trust scholarship tends to group them into three (overlapping) categories, namely, whether an actor is deemed competent (‘ability’), concerned with others’ interests (‘benevolence’) and as conducting themselves honestly and with propriety (‘integrity’) (Mayer et al., 1995). Empirical studies of trust in scientists have often focussed their analysis on factors falling within this Ability, Benevolence, Integrity (ABI) model of trustworthiness (e.g. Hendriks et al., 2015; Reif and Guenther, 2022; Wintterlin et al., 2022), although sometimes employing rather different terms (e.g. ‘warmth’, ‘empathy’ and ‘honesty’; Fiske and Dupree, 2014), and introducing additional factors, such as openness (Besley et al., 2021).

Studies have shown that people generally appraise scientists’ competence as higher than their benevolence or integrity, and the latter two as higher than their openness (Besley et al., 2021). In the words of Fiske and Dupree (2014), scientists are viewed as ‘competent’ but not ‘warm’. Studies also suggest that perceptions of scientists’ competence are sometimes more closely associated with trust than are perceptions of their benevolence and integrity (Besley and Tiffany, 2023: Study 2). When asked, via open-ended survey questions, to identify the reasons for their trust in scientists, people have been found to list as the primary consideration measures tapping ‘expertise’ and ‘competence’ over measures tapping ‘integrity’ and ‘benevolence’ (Bromme et al., 2022; Purvis et al., 2021).

However, other studies suggest that people’s trust in scientists reflects considerations beyond competence. Thus, some analyses report that people’s trust in scientists and scientific information is equally or even primarily shaped by perceptions of benevolence: measured as a perceived concern of scientists with the public interest (Besley and Tiffany, 2023: Studies 1 and 3; Critchley, 2008), by whether scientists are seen to share people’s interests (Eiser et al., 2009) or values (Earle et al., 2010), or by whether scientists are perceived to hold other-regarding motivations over self-regarding motivations (Benson-Greenwald et al., 2023). People’s trust in scientists has also been found to reflect qualities of perceived objectivity and truthfulness (Hunt and Frewer, 1999; Safford et al., 2021), along with the perceived openness and transparency of scientific practices (Landrum et al., 2018; Reif and Guenther, 2022).

Given these results, we anticipate that people’s trust in scientists will be associated most closely with evaluations of scientists’ ability or competence; but that trust will also substantially reflect evaluations of scientists’ perceived benevolence and integrity (*H1*).

*H1: People’s trust in scientists will be more closely associated with perceptions of scientists’ competence than with perceptions of scientists’ benevolence and integrity. Nonetheless, the latter will still be substantively associated with trust.*

People’s trust judgements may rest not only on scientists’ general traits and qualities, but also on appraisals of these actors’ behaviour or relationships with decision-makers. Such behaviours and relationships are particularly likely to affect individuals’ trust judgements during public health emergencies or crises involving social risk, when these aspects of scientists’ work become more publicly noticeable and thus salient. During the coronavirus pandemic, two aspects of scientists’ roles attracted particular public attention, and thus might be thought potentially relevant to individual trust judgements. The first was the proximity in many countries of senior scientific advisers to elected politicians and governments (Lavazza and Farina, 2020). While this proximity enabled a ready infusion of scientific expertise into policy decisions, it also raised questions about the independence and politicisation of scientific advisors (Koch and Durodié, 2022; Weingart et al., 2022). There is evidence that some scientific advisory bodies established in response to the coronavirus – the COVID-19 Scientific Council in France, for example – were seen by many citizens as not being independent of government (Schultz and Ward, 2021). Moreover, there is evidence that individual distrust of scientific agencies like the US Centres for Disease Control and Prevention in part reflected perceived politicisation of its information and guidance (Purvis et al., 2021). Accordingly, we anticipate that people’s trust in scientists will be shaped by judgements about the independence of scientists from partisan political actors.

*H2: People’s trust in scientists will be higher if they perceive scientists to be more, rather than less, independent of political actors.*

The second aspect of scientists’ role during the coronavirus pandemic that potentially shaped individuals’ trust is the way these actors were seen to behave and in particular their perceived observance of official coronavirus restrictions. Public trust in official actors has been found to have declined after cases where high-profile individuals were revealed to have broken official coronavirus rules (Fancourt et al., 2020). Moreover, in Britain at least, there were cases where prominent scientists were shown to have breached these rules.^2^ Although a study conducted across six European countries found people more likely to believe that politicians will break official coronavirus rules than will scientists, more than one quarter (28%) also believed that scientists ‘ignore rules and procedures’ (Peritia, 2022). Accordingly, we anticipate that people’s trust judgements are likely to draw on perceptions of whether scientists observe collective coronavirus rules.

*H3: People’s trust in scientists will be higher if they perceive scientists to have observed coronavirus rules than if they perceive them to have broken those rules.*

We therefore hypothesise that people’s trust in scientists is likely to be based not only on perceptions of scientists’ general traits (*H1*), but also on perceptions of scientists’ position within the decision-making process *(H2)* and behaviour (*H3*).

Important public health information is often provided by a mixture of non-partisan and partisan sources. We therefore compare the bases of people’s trust in scientific experts and politicians to identify whether trust in each reflects similar or distinct considerations. To inform this comparison, we observe that partisan actors play – by virtue of their elected status – a formal representative role, while unelected scientists do not. This leads us to hypothesise that people’s trust in politicians will rest more strongly on evaluations of benevolence (a concern with others, and related to the representative role) than will their trust in scientists. However, reflecting the anticipated importance of competence evaluations to people’s evaluations of scientists (*H1*), we also hypothesise that people’s trust in scientists will be more strongly shaped by competence appraisals than will their trust in politicians. When it comes to integrity, we have noted previous studies showing that people’s trust in scientists partly reflects judgements of these actors’ objectivity, truthfulness and openness, characteristics which have also been shown to matter for people’s trust in politicians (Martin et al., 2020). Accordingly, we hypothesise that appraisals of integrity will be equally closely associated with people’s trust in scientists and in politicians.

*H4: Compared to their trust in politicians, people’s trust in scientists will be*:
  (a) *Less strongly associated with evaluations of benevolence*
  (b) *More strongly associated with evaluations of competence*
  (c) *Equally strongly associated with evaluations of integrity.*

## 2. Data and methods

To test these hypotheses, we collected data on people’s trust in different sources of coronavirus-related information.^3^ Data were obtained from two online surveys conducted by Ipsos-MORI in February 2022 among samples of the British and US populations aged 18+ drawn from the company’s online panels. By extending coverage across two countries, we can test for any variations in the factors associated with people’s trust in scientists (and politicians) across national contexts.^4^ To ensure the representativeness of samples, quotas were set on age, gender, region and working status. The distributions are weighted to the known offline population proportions for age, working status and social grade within gender and region (for the British sample) and for age within gender, working status, household annual income and region (for the US sample).^5^ The total number of respondents was 1501 in Britain and 1499 in the United States.

The surveys collected two types of data appropriate to analysing the factors associated with people’s trust judgements. The first comprised discrete choice data gathered through a conjoint experiment embedded within the surveys.^6^ Conjoint designs are an effective way of identifying the causal effects of specific features of an object characterised by multiple attributes (Hainmueller et al., 2014). The use of a conjoint experiment enabled us to simultaneously test the effects on trust of different features of scientists (and politicians), their role and their work, and to compare the magnitudes of these associations. Data from the conjoint experiment are used to test *H1, H2* and *H4*. The second type of data comprised a set of self-reported beliefs – or observational data – derived from survey questions on scientists’ (and politicians’) traits and behaviours. These data were used to test *H3*, as well as contributing to tests of *H1*.

### Choice data

The choice data derive from a conjoint experiment. We split our British and US samples into two groups (~750 respondents in each, with each split-sample being separately weighted). Respondents in each group were presented with pairs of a single actor – either a scientist advising government on COVID-19 or a politician (a government minister in Britain; a state governor in the United States) – and asked to choose which one they would trust more to provide reliable information about COVID-19.^7^ The actors within each pair varied across a set of attributes,^8^ and each respondent was asked to make four pairwise choices relating to their trust in either scientists or politicians. We assume that these choices will reflect the attributes presented by the actors within each pair.

Each actor was characterised by eight separate attributes, which we selected to cover the features of scientists identified in previous studies, along with key aspects of their work during the coronavirus pandemic. These attributes covered scientists’ (and politicians’) perceived traits (competence, benevolence, honesty, transparency and representativeness), position in the policy process (independence of decisions), communication style (complexity of language used) and propensity to balance scientific evidence with wider social and economic values (which we operationalised in terms of considering the needs of the business sector). The eight attributes, and the key quality associated with each, were:

- *Competence*. Actor’s quality of work (high/average/low).
- *Transparency*. Making public (all/some/none of) the data and information used in their work.
- *Representativeness*. Being (very/a bit) in touch or out of touch with everyday life and people like yourself.
- *Benevolence*. Being concerned (very/somewhat/not very) with ordinary peoples’ lives.
- *Honesty*. Admitting (always/sometimes/rarely) when the evidence does not support previous claims.
- *Communication*. Use of language when presenting information (use of technical/easy-to-understand language).
- *Independence*. Adjustment of scientific evidence to politicians’ beliefs (considers evidence but adjusts decisions/considers evidence alone and does not adjust decisions).
- *Values*. Balance of scientific evidence with needs of business (balances evidence with needs of business/focuses only on evidence and does not consider needs of business).

**Figure 1.**
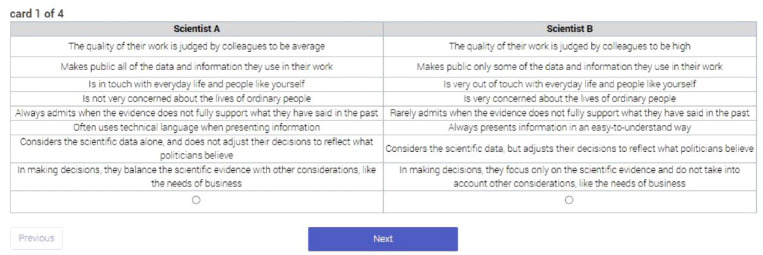
Example of the presentation of the conjoint choice task.

A full description of the attributes and the levels within them – which, to facilitate comparisons, were both worded almost identically across scientists and politicians – is provided in Supplemental Material Appendix 1, while an example of the conjoint presentation is provided in Figure 1. In analysing the choice data, we follow Hainmueller et al. (2014) and cluster responses by individual. We follow the guidance of Leeper et al. (2020) and calculate marginal means for each attribute, which enables us to identify the probability of choices associated with an attribute at all its levels rather than relative to a designated baseline.

### Observational data

We complement the choice data with observational data derived from survey questions (full details of which are provided in Supplemental Material Appendix 2). Our survey measures were designed to tap three particular sets of evaluations. The first was whether people feel scientists (and politicians) comply with collective coronavirus rules. We assume that perceptions of rule observance will primarily shape people’s trust in the actor whose behaviour is being reviewed (i.e. trust in X will primarily be shaped by appraisals of X’s behaviour). However, the close working relations of senior scientists and politicians during the coronavirus pandemic raises the potential that behavioural transgressions by one actor might also shape people’s trust in the other. In our empirical modelling, we explore the evidence for any such ‘transfer’ effects from judgements about the behaviour of one actor to trust in another actor.

The second evaluation related to people’s appraisals of scientists’ competence. Here, we complement our measure of competence in the conjoint experiment by fielding a different form of competence appraisal, namely, a survey item tapping perceptions of scientific accuracy in predicting coronavirus infections. Note that, since there was no direct analogue of this measure that could readily be applied to politicians, we did not field a comparable survey question tapping politicians’ competence. However, as in the case of rule-compliance, we anticipate that people’s appraisals of scientists’ competence might shape their trust not only in scientists but also in politicians. Thus, when it comes to modelling people’s trust in politicians, we explore what role might be played by perceptions of scientists’ predictive accuracy.

The third evaluation related to how well scientists (and politicians) were perceived to represent individual values, a factor that, as already noted, has been identified as an important predictor of trust (see Earle et al.’s (2010) ‘value similarity’ model of trust). We measured representation of values by reference to how far the perceived policy positions of scientists approximated the ideal position of individual survey respondents. These two spatial positions were gauged using twin scales (derived from the British Election Study) that captured different aspects of the normative debates over coronavirus lockdowns. Both scales were anchored at one end by the desirability of reducing coronavirus infections. At the other end, the first scale was anchored by the desirability of protecting the economy, while the second scale was anchored by the desirability of protecting people’s freedoms. Respondents identified their own ideal position on each of these scales, and then did the same for the positions they perceived scientists (and politicians) to represent. Representation (or ‘shared values’) was then calculated as the distance on each scale between the individual’s own position and the positions they ascribed separately to scientists and to politicians.

In modelling the observational data, we include control variables measuring various factors identified in previous studies as closely associated with trust in scientists. We measure belief in science (Wintterlin et al., 2022) via a validated six-item battery of measures tapping individual beliefs that science constitutes an appropriate form of knowledge (indicative item: ‘All the tasks human beings face can be solved by science’; Farias et al., 2013). We measure fear of the coronavirus (Dryhurst et al., 2020; Schneider et al., 2021) via two survey items tapping perceived personal and community risk.^9^ We also measure partisanship (close relationships between partisanship and trust in scientists have been identified in the United States in particular, with Republicans found to be less trusting in scientists than Democrats; Hamilton and Safford, 2021; Krause et al., 2019), orientations to authority (authoritarian individuals have been found less supportive than liberals of the role of science in public decision-making: Gauchat, 2015), religious beliefs (people holding religious beliefs have been found to be less trusting of scientists than their non-religious counterparts; Krause et al., 2019), ethnicity (Craig et al., 2020; Stead et al., 2021), education, socio-economic status (Stead et al., 2021), age and gender.

The dependent variables in our models of the observational data comprise trust based on the following survey question: ‘How much, if at all, do you trust each of the following when it comes to providing information about COVID-19?’. Respondents were provided with a list of sources, including ‘scientific and medical experts’, and either ‘government ministers’ (in Britain) or ‘the federal government’ (in the United States). Trust was measured on a 0 (no trust at all) to 10 (full trust) scale, and models are estimated using ordinary least-squares regression. ‘Don’t know’ responses are set to missing, while to facilitate comparisons continuous independent variables are normalised (on a 0–1 scale).

In analysing the choice and observational data, we pool observations across the British and US samples reflecting the fact that, as we demonstrate below, our results are substantially similar across contexts. Where there are variations in the results between the two samples, we highlight these in the text and report them more fully in Supplemental Material Appendix 3 (choice data) and Supplemental Material Appendix 4 (attitudinal data).

## 3. Results

### Choice data

**Figure 2.**
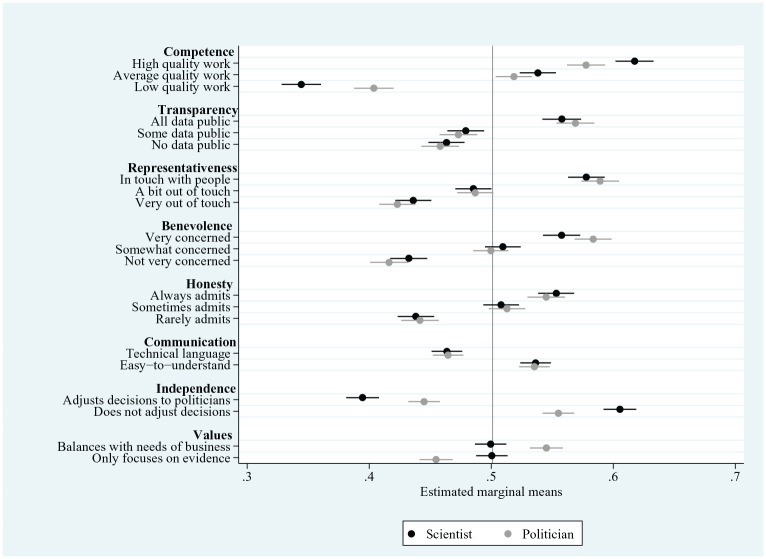
Effects of source attributes on trust – choice data. Data are pooled British/US samples. Whiskers show 95% CIs.

We begin by analysing the results from the conjoint experiment. These results are laid out in full in Supplemental Material Appendix 5, and summarised in Figure 2, which shows the marginal means for each level of the eight assessed attributes (values above the 0.5 level indicate the attribute level is associated with a higher level of trust; values below the 0.5 level indicate the attribute level is associated with a lower level of trust).

We find all but one of the eight attributes to be significantly associated with people’s trust in scientists. The strongest association with trust is for competence; the effect of a scientist’s work being described as being of ‘high’ quality rather than ‘low’ quality is to increase levels of trust by 27 percentage points (from 0.34 (95% confidence intervals (CIs): 0.33, 0.36) to 0.62 (95% CIs: 0.60, 0.63)), averaging across the levels of the other attributes. We also find people’s trust in scientists to be substantively associated with their perceived relations with the wider public. A scientist presented as representative (‘in touch with everyday life and people like yourself’) attracts a 14 percentage-point higher level of trust than a scientist presented as unrepresentative (‘not in touch’ with everyday life and people). A similar effect on trust is seen for benevolence (a scientist presented as ‘very concerned with the lives of ordinary people’ as opposed to one ‘not very concerned’ with ordinary people’s lives). The results also highlight a significant effect on trust of evaluations of integrity. Thus, a scientist presented as being fully transparent attracts a 10 percentage-point higher level of trust than a scientist presented as not transparent at all.^10^ A similar effect on trust is found for honesty; a scientist presented as always admitting to mistakes is 12 percentage points more trusted than one who rarely admits to such mistakes.

Collectively, these results provide support for *H1*. We find that, while people’s trust in scientists is most strongly shaped by judgements of competence, it also substantively reflects judgements of benevolence and integrity. The results from the conjoint data also suggest that people’s trust in scientists reflects not only scientists’ perceived qualities and traits, but also their location within the decision-making process and in particular their independence from politicians. Where scientific decisions are presented as being driven by the science alone and as independent from political considerations, scientists attract a significantly higher level of trust (marginal mean: 0.61; 95% CIs (0.59, 0.62)) than where those decisions are presented as adjusted to reflect politicians’ views (marginal mean: 0.40; 95% CIs (0.38, 0.41)). Yet, trust does not appear to be affected by whether scientists’ decisions balance the scientific evidence with wider considerations, such as the needs of business.

We therefore find support for *H2*: trust in scientists is lower where partisan considerations are seen to interfere with scientific decisions. Yet, there does not appear to be any negative impact on trust where scientists’ decisions are confined to the scientific evidence and do not also take account of non-scientific considerations, such as the needs of the commercial sector.

The conjoint data also enable us to compare the factors shaping people’s trust in scientists with the factors shaping their trust in politicians. We begin by noting that trust in scientists rests more heavily than does trust in politicians on evaluations of competence. When it comes to trust in scientists, we have pointed to the large difference (of 27 percentage points) associated with presenting scientific work as being of either low or high quality. Trust in politicians is similarly greater when government actors’ work is presented as high quality rather than low quality; but the increase in trust is less substantial at 18 percentage points (marginal mean of ‘high quality work’ = 0.58; 95% CI (0.56, 0.59)); of ‘low quality work’ = 0.40; 95% CI (0.39, 0.42)). This result confirms our expectation in *H4b*.

We also find that evaluations of integrity have equivalently sized effects on people’s trust in both scientists and politicians. We have seen that presenting a scientist as honest (‘always admitting when the evidence does not support past claims’) increases trust by 12 percentage points and as transparent (‘Makes public all of the data and information they use in their work’) by 10 percentage points. The equivalent increases in trust in the case of politicians are 10 and 11 percentage points. We therefore find confirmation for *H4c*.

Yet, there is little evidence that perceived benevolence has a greater effect on people’s trust in politicians than on their trust in scientists. We have already seen that a scientist presented as benevolent (‘very concerned’ about the lives of ordinary people) attracts a 13 percentage point higher trust rating than their non-benevolent counterpart (‘not very concerned’ about ordinary people). The equivalent gap for politicians is only a little higher at 16 percentage points. We also observe that a similar concept – namely, representativeness (being presented as ‘in touch’ versus ‘out of touch’ with everyday life) – has a similar effect on people’s trust in scientists (where it is associated with an increase in trust of 14 percentage points) and politicians (where the increase is 17 percentage points). We therefore fail to confirm *H4a*, that perceptions of benevolence have greater effects on people’s trust in elected partisan actors than on their trust in unelected scientific actors.

The only characteristic where there is a discernible difference in the effect on people’s trust in scientists and politicians concerns decision-making that reflects a wider set of (economic) interests. We have seen that people’s trust in scientists is not shaped by whether scientific decisions are presented as incorporating the needs of business. Yet we find that a politician whose decisions are presented as drawing solely on the scientific evidence is less trusted than their counterpart whose decisions are presented as incorporating business needs.

Finally, we note that, in the main, the results from the conjoint experiment apply fairly consistently across the British and American samples. Any differences in the effects of specific attributes on trust between the two samples are not substantial (for details, see Supplemental Material Appendix 3).

### Observational data

**Figure 3.**
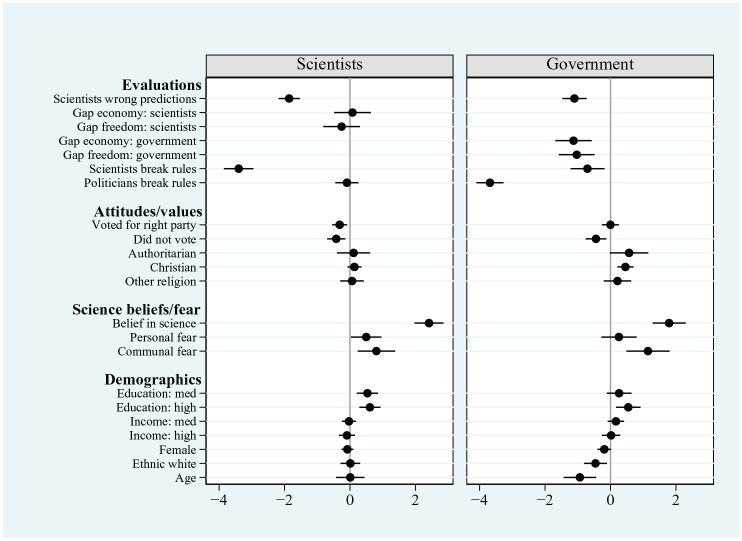
Factors associated with trust – observational data. Data are pooled British/US samples. Dots show point estimates; Whiskers show 95% CIs.

We now turn to the results of our modelling of the observational data, which are presented in full in Supplemental Material Appendix 6 and summarised in Figure 3 (which presents OLS coefficients for each of the independent variables on trust in both scientists and government actors). The results show that people’s trust in scientists is closely associated with competence judgements. Among people who strongly agree that scientists have made faulty, rather than accurate, predictions about coronavirus infections, trust in scientists is on average almost 1.9 points lower (on an 11-point scale; *p* < .01) than among people who strongly disagree that these predictions have often been wrong. This highlights the role of competence judgements in people’s trust in scientists, providing additional confirmation of *H1*.

People’s trust in scientists is also closely associated with their appraisals of scientists’ behaviour; levels of trust are significantly lower (by more than 3.3 points on an 11-point scale; *p* < 0.01) if individuals perceive scientists never to have followed official coronavirus rules than if scientists are perceived nearly always to have followed these rules. This close association of appraisals of scientists’ behavioural compliance with trust provides support for *H3*.

Yet, we do not find any significant effect on trust of whether scientists are perceived to represent individuals’ own views on the coronavirus lockdown. Whether the lockdowns are framed in terms of their effect on the economy or on personal freedoms, we find that scientists’ proximity to individual views is not significantly associated with trust.

When it comes to individuals’ broader attitudes – fielded as control variables in the models – we find trust in scientists to be significantly higher among people holding positive attitudes about the role of science (measured by the six-item beliefs in science scale) than among their more sceptical counterparts. We also find that trust in scientists is higher among people expressing fear of the coronavirus, both personally and at the community level.^11^ Partisanship is significantly associated with people’s trust in scientists, with lower levels of trust among individuals who voted for right-wing parties than among those who voted for left-wing parties. However, this effect is solely driven by participants in the US sample (see the separate results for Britain and the United States in Supplemental Material Appendix 4; for a discussion, see Hegland et al., 2022).^12^ By contrast, among the British sample, electoral support for right-wing parties (the Conservative and Brexit parties) is not significantly associated with trust in scientists.^13^ Other factors – such as holding authoritarian attitudes and religious views – are not significantly associated with people’s trust in scientists. Neither are individuals’ demographic characteristics, with the exception of education which is positively associated with people’s trust in scientists.

To what extent are the factors associated with people’s trust in scientists similarly associated with their trust in politicians? The results presented in Figure 3 show some similarities in the bases of people’s trust in the two actors. Politicians who are seen to break coronavirus rules are associated with lower rates of trust in the government just as much as perceived rule-breaking scientists are with trust in scientists. On this measure at least, breaches of integrity appear equally damaging for people’s trust in expert and partisan sources of health-related information (reinforcing the results from the conjoint data, and providing additional confirmation of *H4c*). Yet we also find some differences in the sources of people’s trust in scientists and politicians. We have already noted that representation of individual values is not significantly associated with people’s trust in scientists. Yet when it comes to trust in politicians, the results presented in Figure 3 show a significant association with such shared values. When government actors are seen as distant from, rather than proximate to, individuals’ own lockdown preferences, there is a clear negative association with trust.^14^

Finally, we note some evidence of the way that people’s evaluations of one actor ‘transfer’ into their trust in the other actor, albeit not in a consistent manner. We find that people’s evaluations of scientists’ ability to predict coronavirus infections are significantly associated not only with their trust in scientists, but also with their trust in politicians. In the case of compliance with coronavirus rules, we similarly find that perceptions of scientists’ behaviour (i.e. whether they observe the rules) are significantly associated not only with people’s trust in scientists but also with their trust in politicians. Yet the reverse does not hold; we find that perceived rule-compliance among politicians is not significantly associated with people’s trust in scientists.

In summary, the observational data confirm that people’s trust in scientists is closely associated with evaluations of these actors’ competence and behaviour. Yet trust does not appear to be shaped by individuals’ judgements about whether scientists share their own values, at least on the merits of coronavirus lockdowns. In this, people’s trust in scientists rests on somewhat different grounds to their trust in politicians.

## 4. Discussion

This study has taken advantage of conditions in which scientific experts have been thrust – willingly or otherwise – into the public gaze to explore the factors associated with people’s trust in them. Because of their high-profile position during the coronavirus pandemic, our study was able to probe a variety of evaluations relevant to public trust, going beyond a narrow set of traits or qualities attached to scientists to include a wider range of behaviours and relationships whose relationship with people’s trust has, to date, gone largely unexplored. The use of a conjoint research design also enabled us to test the simultaneous effects on trust of a wider set of attributes of scientists than attempted in most previous studies.

Our results confirm the importance to people’s trust in scientists of competence evaluations. Across the different measures of competence employed in our research design – choice-related scenarios of low v high quality scientific work or attitudinal measures of scientists’ predictive accuracy – we found people’s evaluations of scientists’ competence to be central to their trust in these actors.

Yet our results also highlight the way people’s trust in scientists is also rooted in evaluations of their benevolence and integrity. Trust is higher when scientists are seen to be in touch with, and concerned by, the needs of ordinary people. We conclude that scientific experts cannot ‘cocoon’ or insulate themselves from wider society; to gain public trust, they need to demonstrate some understanding of, and empathy with, the wider population (for similar results, see Hunt and Frewer, 1999). People’s trust is also shaped by considerations of integrity, for example, by evaluations of whether scientists abide by collective behavioural rules, and by presentations of scientists as transparent and honest or not. If the effects of scientists’ transparency and honesty on trust identified in this study are rather modest – compared to the findings of previous studies (Hunt and Frewer, 1999; Landrum et al., 2018; Reif and Guenther, 2022) – this may reflect our choice of research design. Conjoint experiments force participants to make choices across a range of considerations rather than against just one or two considerations as has often been the case in previous research designs. Testing multiple attributes of an actor simultaneously through a conjoint design offers a way of capturing individual judgements across different considerations in a way that is arguably more sensitive to the way that individuals form evaluations in reality.

Our results also highlight that people’s trust in scientists (in the context of a national emergency at least) rests not only on these actors’ traits and qualities, but also on their behaviour and relations with other political actors. We found levels of trust to be depressed when scientific decisions were characterised as being ‘politicised’ through deference to partisan considerations. This suggests that, even when scientists are centre-stage in government-sponsored public health press conferences and information presentations, they should maintain their distance from partisan actors. Our results show that any blurring of these boundaries entails a price to public trust. Of course, scientists cannot always prevent their role being politicised, if their findings and factual statements are misreported or misinterpreted by individuals intent on discrediting their work (Druckman, 2017). What we point to here is a different concern; the danger – for public trust – of scientific findings being seen as compromised by incorporating, or even deferring to, political considerations, and thus as lacking independence and rigour (see also Bolsen et al., 2014).

At the same time, our analysis of the bases of people’s trust in scientists and politicians suggests areas where the former might have some latitude to behave in ways apparently denied to the latter. While, as noted, trust in scientists partly rests on whether people view these actors as in touch and concerned with members of the public, it does not appear to rest on whether scientists’ messages match individuals’ personal values. Nor are scientists deemed untrustworthy if their decisions fail to reflect other social goods, such as the needs of business. By contrast, both judgements are important for people’s trust in politicians. Individuals evaluate (i.e. trust) political actors in terms of whether they represent individual values and wider social demands. In addition, we point to the way in which people’s trust in politicians as information sources reflects not only their evaluations of politicians’ own qualities and behaviours but also, in some cases, on their evaluations of scientists’ qualities and actions. Thus, scientists who are seen to break important collective rules, and to issue inaccurate scientific predictions, depress people’s trust not only in themselves but also in politicians. This suggests a cautionary message for governments in co-opting scientific expertise to the decision-making process. While ‘bringing scientists in’ might bolster the credibility of politicians’ pronouncements, any public perceptions of rule-breaking or incompetence on the part of scientists appear to flow over into more negative trust judgements of politicians themselves.

We recognise that these findings arise from a study conducted at a particular time and context, which may well influence the construction of individuals’ trust judgements. We also recognise that the findings are drawn from samples in just two countries. However, we point to the essential similarity of the results – from the conjoint experiment at least – in Britain and the United States as tentative evidence that these findings might generalise beyond these two cases to populations in other advanced democracies. Nonetheless, we would welcome replication studies carried out in different time periods and contexts – for example, exploring people’s attitudes to different types of scientist and politician – to validate this supposition and to confirm the wider applicability of our findings.

## 5. Conclusion

The coronavirus pandemic originating in 2020 emphasised just how much ‘trust matters’, and the relevance in particular of people’s trust in scientific experts. This makes it imperative that we understand the factors contributing to scientists’ trustworthiness in the public mind. This study has built on earlier analyses of popular evaluations of scientific experts to identify how various attributes of scientists and their work might contribute to public judgements of trust. Our findings are instructive for the ways in which scientific experts might operate and position themselves to maximise public trust, and thus to stimulate individuals’ acceptance of key health-related information and guidance. Overall, we stress that while perceptions of scientific competence are central to public judgements of trust, these judgements also reflect a wider set of considerations. The work of scientists has broad implications for wider society – particularly during public emergencies and crises – and people’s trust reflects how closely they perceive scientists to be attuned to, and concerned by, citizens’ needs. We also stress that, while scientists’ incorporation into government processes holds advantages in bringing scientific evidence closer to official policy-making, it also comes with risks. We suggest that while scientific experts need not overly worry about the Scylla of coordinating their guidance with other sectoral needs, they should steer clear of the Charybdis of any politicisation of their role.

## Supplemental Material

sj-docx-1-pus-10.1177_09636625241253968 – Supplemental material for ‘Follow the science’: Popular trust in scientific experts during the coronavirus pandemic Supplemental material, sj-docx-1-pus-10.1177_09636625241253968 for ‘Follow the science’: Popular trust in scientific experts during the coronavirus pandemic by Ben Seyd, Joseph A Hamm, Will Jennings, Lawrence McKay, Viktor Valgarðsson and Meridith Anness in Public Understanding of Science

## Notes

1. We distinguish this approach to trust from an approach that sees trust as manifested in a willingness to make oneself vulnerable to another actor, a more behavioural form of trust.

2. The most public example of this concerned Professor Neal Ferguson of Imperial College, who stepped down as a government scientific adviser in May 2020 having been caught breaking official lockdown rules.

3. Project details are available at: https://research.kent.ac.uk/information-trust/.

4. The populations of our two study countries manifested similar levels of trust in scientists, which have declined somewhat since the period before the coronavirus pandemic. Among both populations, moreover, there were low levels of trust in politicians immediately prior to the pandemic and these levels subsequently declined further (for the United Kingdom, see Ipsos, 2023; for the United States, see Kennedy and Tyson, 2023). While patterns of trust were similar between the two countries, the United States saw a more politicised reaction to the role of scientists during the coronavirus pandemic (Stroebe et al., 2021). These features lead us to anticipate broad consistency across the two cases in the factors associated with trust in scientists, with the exception of partisanship where we anticipate stronger associations with trust in the United States than in Britain.

5. The US sample in particular is not representative on ethnicity. However, our previous work identifies limited differences in the factors associated with trust across different ethnic groups.

6. The conjoint experiment was fielded before the collection of observational data so that respondents’ choices were not influenced by any prior expressed attitudes.

7. We specified scientists advising government and government ministers/state governors as these seemed the most relevant actors in the context of the coronavirus. However, we recognise that people’s trust in scientists may vary depending on the sector (Castell et al., 2014) and field (Sonmez et al., 2023) of scientific work. It would be valuable to replicate the present exercise on people’s trust in scientists working in different sectors and fields, and indeed on different types of political actors.

8. Technical facilities of the survey company meant we could only randomise the order in which particular actor profiles were presented to participants, not the order or levels of specific attributes. Strictly speaking, this violates the requirement that choices are statistically independent of the presented profiles, which requires randomisation of attributes within profiles (Hainmueller et al., 2014). However, there is little evidence that the randomisation procedure in our study compromised the results. We point to the fact that, while ‘competence’ (the first presented attribute; see Figure 1) has the largest individual effect on people’s trust in scientists (Figure 2), the second largest effect is for ‘independence’ (the seventh out of eight presented attributes). We also note that ‘competence’ has no greater effect on people’s trust in politicians (Figure 2) than some other attributes featuring lower down the list of presented attributes.

9. The item measuring personal fear was drawn from the British Election Study.

10. Note that moderate transparency (the scientist described as making public ‘only some of the data and information they use in their work’) is still associated with lower levels of trust (i.e. the marginal mean for this attribute falls below the 0.5 level); only full transparency (making public ‘all of the data’) is associated with higher trust (i.e. above the 0.5 level).

11. The importance for people’s trust in scientists of judgements about scientists’ competence and behaviour, along with attitudes towards the role of science and coronavirus fear, is shown in the effects of the ‘Evaluations’ and ‘Science beliefs/fear’ blocks of variables, which, respectively, account for 0.36 and 0.26 of the variance in trust (measured by the *R*^2^ figure for each block of measures). By contrast, far less of the variance is explained by the factors measured in the ‘Attitudes/values’ and ‘Demographics’ blocks. For details, see Supplemental Material Appendix 6.

12. Although this effect does not appear to solely reflect ideology, as trust in scientists is also lower among Americans who report not having voted at all, compared to those voting for left candidates.

13. We also note that, when it comes to predicting people’s trust in government (discussed further below), beliefs in science are not a significant predictor of trust in Britain, although they are in the United States. This provides further confirmation of the extent to which, in the United States, science has been politicised (see Gauchat, 2015).

14. As shown in Supplemental Material Appendix 4, the association with trust in the British case arises primarily in relation to the gap on the lockdown-personal freedom scale, while in the US case, the gap arises primarily in relation to the gap on the lockdown-economy scale.
