# Supplementary material for: ‘Follow the science’: Popular trust in scientific experts during the coronavirus pandemic
Source: Public Underst Sci. 2024 Jun 12;34(1):2–18. doi: 10.1177/09636625241253968 (PMC11673289; doi:10.1177/09636625241253968)
Supplement: sj-docx-1-pus-10.1177_09636625241253968 – Supplemental material for ‘Follow the science’: Popular trust in scientific experts during the coronavirus pandemic [file sj-docx-1-pus-10.1177_09636625241253968.docx]

**‘FOLLOW THE SCIENCE’: POPULAR TRUST IN SCIENTIFIC EXPERTS DURING THE CORONAVIRUS PANDEMIC**

Ben Seyd, Joseph A Hamm, Will Jennings, Lawrence McKay, Viktor Valgarðsson and Meridith Anness

**Supplemental material**

[APPENDIX 1: Attributes/levels in conjoint study 2](#_Toc161150688)

[APPENDIX 2: Details of variables used in regression models 3](#_Toc161150689)

[APPENDIX 3: Results of conjoint data – separate country samples 6](#_Toc161150690)

[APPENDIX 4: Results of observational data – separate country samples 8](#_Toc161150691)

[APPENDIX 5: Full results of conjoint data models 10](#_Toc161150692)

[APPENDIX 6: Full results of regression models of observational data – pooled country samples 12](#_Toc161150693)

# APPENDIX 1: Attributes/levels in conjoint study

| Attribute |  | | Levels |
| --- | --- | --- | --- |
|  |  | |  |
| *Competence* |  | The quality of their work is judged by colleagues to be high  The quality of their work is judged by colleagues to be average  The quality of their work is judged by colleagues to be low | |
|  |  |  | |
| *Transparency* |  | Makes public all of the data and information they use in their work  Makes public only some of the data and information they use in their work  Makes public none of the data and information they use in their work | |
|  |  |  | |
| *Representativeness* |  | Is in touch with everyday life and people like yourself  Is sometimes a bit out of touch with everyday life and people like  yourself  Is very out of touch with everyday life and people like yourself | |
|  |  |  | |
| *Benevolence* |  | Is very concerned about the lives of ordinary people  Is somewhat concerned about the lives of ordinary people  Is not very concerned about the lives of ordinary people | |
|  |  |  | |
| *Honesty* |  | Always admits when the evidence does not fully support what they have said in the past  Sometimes admits when the evidence does not fully support what they have said in the past  Rarely admits when the evidence does not fully support what they have said in the past | |
|  |  |  | |
| *Communication* |  | Often uses technical language when presenting information  Always presents information in an easy-to-understand way | |
|  |  |  | |
| *Independence* |  | Considers the scientific evidence, but adjusts their decisions to reflect what politicians believe*  Considers the scientific evidence alone, and does not adjust their  decisions to reflect what politicians believe* | |
|  |  |  | |
| *Values* |  | In making decisions, they balance the scientific evidence with other considerations, like the needs of business  In making decisions, they focus only on the scientific evidence and do not take into account other considerations, like the needs of business | |
|  |  | |  |

* The version for politicians reads “… what other politicians believe”.

# APPENDIX 2: Details of variables used in regression models

| **Variable** | **Coding** | **Mean**  **(proportions)** | **SD** | **N** |
| --- | --- | --- | --- | --- |
|  |  |  |  |  |
| *Trust* |  |  |  |  |
| How much, if at all, do you trust each of the following when it comes to providing information about COVID-19? | | | | |
| Government ministers/federal government | 0 (no trust at all) to 10 (full trust) | 4.29 | 2.96 | 2,947 |
| Scientific and medical experts |  | 6.64 | 2.78 | 2,954 |
|  |  |  |  |  |
| *Rule following* |  |  |  |  |
| How often, if at all, do you think the following groups tend to follow official guidance and rules designed to minimise the spread of the coronavirus? | | | | |
| Government ministers/state governors | 0 (nearly always) to 1 (never) | 0.47 | 0.28 | 2,800 |
| Scientific and medical experts |  | 0.26 | 0.25 | 2,776 |
|  |  |  |  |  |
| *Scientists’ predictive ability* | | | | |
| The predictions about the number of coronavirus infections made by scientists advising the government have often been wrong |  |  |  |  |
|  | 0 (strongly disagree) to 1 (strongly agree) | 0.54 | 0.31 | 2,740 |
|  |  |  |  |  |
| *Representation of values* | | | | |
| Some people think that the government should do everything it can to reduce the number of coronavirus infections, even if it damages the economy. Others think that the government should do everything it can to protect the economy, even if it increases the number of coronavirus infections. Whereabouts on this scale would you place the beliefs of each of the following, where 0 is they strongly believe that coronavirus infections should be reduced even if this damages the economy and 10 equals they strongly believe the economy should be protected even if this increases coronavirus infections? Yourself/the government/scientific and medical experts | | | | |
| Gap: Personal – Scientists^1^ | 0 (no gap) to 1 (large gap) | 0.17 | 0.22 | 2,801 |
| Gap: Personal – Govnt |  | 0.20 | 0.26 | 2,812 |
|  |  |  |  |  |
| Some people think that the government should do everything it can to reduce the number of coronavirus infections, even if this means restricting people’s freedoms. Others think that the government should do everything it can to protect people’s freedoms, even if this means increasing the number of coronavirus infections. Whereabouts on this scale would you place the beliefs of each of the following, where 0 is they strongly believe that coronavirus infections should be reduced even if this restricts people’s freedoms and 10 equals they strongly believe people’s freedoms should be protected even if this increases coronavirus infections? | | | | |
| Gap: Personal – Scientists^1^ | 0 (no gap) to 1 (large gap) | 0.16 | 0.22 | 2,813 |
| Gap: Personal – Govnt |  | 0.20 | 0.26 | 2,820 |
|  |  |  |  |  |
| *Beliefs in science and scientists* | |  |  |  |
| Summed scale (α=0.89) of responses to 6-item belief in science measures^2^ | | | | |
|  | 0 (low belief in science) to 1 (high belief in science) | 0.58 | 0.24 | 2,957 |
|  |  |  |  |  |
| *Coronavirus fear* | | | | |
| How worried, if at all, are you about you catching and becoming seriously ill from COVID-19? | | | | |
|  | 0 (not at all worried) to 1 (extremely worried) | 0.50 | 0.31 | 2,940 |
| How worried, if at all, are you about the coronavirus situation in: The locality where you live/the country as a whole? (Summed scale; α=0.90) | | | | |
|  | 0 (not at all worried) to 1 (extremely worried) | 0.55 | 0.26 | 2,954 |
|  |  |  |  |  |
| *Partisanship* |  |  |  |  |
| Vote in 2019/2020 national election | |  |  |  |
|  | 1=Other party | (47%) |  | 2,713 |
|  | 2=Right: Conservative/Brexit (Bri) Republican (US) | (35%) |  |  |
|  | 3=Did not vote | (18%) |  |  |
|  |  |  |  |  |
| *Authoritarianism* |  |  |  |  |
| Summed scale (α=0.76) of responses to 6-item libertarian-authoritarian scale^3^ | | | | |
|  | 0 (libertarian) to 1 (authoritarian) | 0.60 | 0.20 | 2,967 |
|  |  |  |  |  |
| *Religious beliefs* |  |  |  |  |
| Do you regard yourself as belonging to any particular religion? | | | | |
|  | 0=no religion | (36%) |  | 2,909 |
|  | 1=Christian | (55%) |  |  |
|  | 2=other religion | (8%) |  |  |
|  |  |  |  |  |
| *Demographics* |  |  |  |  |
| Ethnic status | 0=non-white, 1=white | 0.88 | 0.33 | 2,939 |
| Education^4^ | 1=low | (11%) |  | 3,000 |
|  | 2=medium | (36%) |  |  |
|  | 3=high | (54%) |  |  |
| Annual household income^5^ | 1=low | (34%) |  | 2,911 |
|  | 2=medium | (37%) |  |  |
|  | 3=high | (29%) |  |  |
| Age | 18 to 92 years (0-1 scale) | 0.42 | 0.22 | 3,000 |
| Gender | 0=Male, 1=Female | 0.53 | 0.50 | 2,968 |
|  |  |  |  |  |

^1^ Gap = Σ [individual position] – [perceived government/scientist position].
^2^ (1) Science provides us with a better understanding of the universe than does religion; (2) We can only rationally believe in what is scientifically provable; (3) Science tells us everything there is to know about what reality consists of; (4) All the tasks human beings face can be solved by science; (5) The scientific method is the only reliable path to knowledge; (6) The only real kind of knowledge we can have is scientific knowledge.

^3^ How much do you agree or disagree with the following statements? (1) Young people today don’t have enough respect for traditional; (2) People who break the law should be given stiffer sentences; (3) For some crimes, the death penalty is the most appropriate sentence; (4) Schools should teach children to obey authority; (5) The law should always be obeyed, even if a particular law is wrong; (6) Censorship of films and magazines is necessary to uphold moral standards. Source: Evans G, Heath A and Lalljee M (1996) Measuring left-right and libertarian-authoritarian values in the British electorate. *British Journal of Sociology* 47(1): 93-112.

^4^ ‘Low’ equates to below GCSE (Britain) and high school diploma and below (US); ‘medium’ equates to GCSE – below degree (Britain) and above diploma – below degree (US); high’ equates to university degree and above (Britain) and associate-level degree and above (US).

^5^ ‘Low’ equates to annual household income before tax of £0-£24,999 (Britain) and $0-44,999 (US); ‘medium’ equates to £25-54,999 (Britain) and $45-89,999 (US); ‘high’ equates to £55,000 and above (Britain) and $90,000 and above (US).

# APPENDIX 3: Results of conjoint data – separate country samples

**(a): Marginal means for attributes of scientists, Britain and US**

F-tests indicate significant differences (p<0.05) between the two samples for the association with trust of: Competence (*x*^2^=30.55), Transparency (*x*^2^=11.05) and Honesty (*x*^2^=8.20).

**(b) Marginal means for attributes of politicians, Britain and US**

F-tests indicate significant differences (p<0.05) between the two samples for the association with trust of: Competence (*x*^2^=12.68), Transparency (*x*^2^=16.84), Representativeness (*x*^2^=10.77), Benevolence (*x*^2^=10.03) and Honesty (*x*^2^=11.15).

# APPENDIX 4: Results of observational data – separate country samples

|  | Trust in scientists | | | | |  | Trust in politicians | | | | |
| --- | --- | --- | --- | --- | --- | --- | --- | --- | --- | --- | --- |
|  | Britain | |  | US | |  | Britain | |  | US | |
| **Evaluations** |  |  |  |  |  |  |  |  |  |  |  |
| Scientists’ predictions often wrong | -1.82** | (.22) |  | -1.77** | (.25) |  | -0.50* | (.25) |  | -1.22** | (.27) |
| Gap on lockdowns-economy: scientists | 0.05 | (.40) |  | 0.27 | (.41) |  | - | - |  | - | - |
| Gap on lockdowns-freedom: scientists | 0.05 | (.40) |  | -0.47 | (.40) |  | - | - |  | - | - |
| Gap on lockdowns-economy: government | - | - |  | - | - |  | -0.57 | (.38) |  | -1.04** | (.39) |
| Gap on lockdowns-freedom: government | - | - |  | - | - |  | -1.35** | (.38) |  | -0.31 | (.38) |
| Scientists don’t follow Covid rules | -3.33** | (.32) |  | -2.92** | (.35) |  | -1.02** | (.37) |  | -0.80* | (.38) |
| Politicians don’t follow Covid rules | -0.31 | (.23) |  | -0.13 | (.32) |  | -3.95** | (.27) |  | -2.10** | (.35) |
|  |  |  |  |  |  |  |  |  |  |  |  |
| **Attitudes and values** |  |  |  |  |  |  |  |  |  |  |  |
| Partisanship^1^ (ref: Voted other) |  |  |  |  |  |  |  |  |  |  |  |
| Voted right party | 0.07 | (.15) |  | -0.94** | (.19) |  | 0.93** | (.18) |  | -1.07** | (.20) |
| Did not vote | -0.16 | (.18) |  | -0.84** | (.23) |  | 0.21 | (.21) |  | -1.03** | (.24) |
| Authoritarian values | -0.43 | (.34) |  | 0.48 | (.39) |  | 0.88* | (.39) |  | 0.53 | (.43) |
| Religion (ref: None) |  |  |  |  |  |  |  |  |  |  |  |
| Christian | 0.20 | (.14) |  | 0.15 | (.17) |  | 0.45** | (.16) |  | 0.40* | (.19) |
| Other religion | 0.53 | (.29) |  | -0.10 | (.25) |  | 0.57 | (.34) |  | -0.11 | (.28) |
|  |  |  |  |  |  |  |  |  |  |  |  |
| **Science beliefs/fear** |  |  |  |  |  |  |  |  |  |  |  |
| High belief in science | 1.74** | (.31) |  | 2.48** | (.33) |  | 0.10 | (.36) |  | 2.66** | (.35) |
| Personal Covid fear | -0.11 | (.33) |  | 0.76* | (.34) |  | -0.07 | (.39) |  | 0.76* | (.37) |
| Communal Covid fear | 1.23** | (.41) |  | 0.80 | (.42) |  | 0.99* | (.47) |  | 1.06* | (.46) |
|  |  |  |  |  |  |  |  |  |  |  |  |
| **Demographics** |  |  |  |  |  |  |  |  |  |  |  |
| Education (ref: low qualifications) |  |  |  |  |  |  |  |  |  |  |  |
| Medium qualifications | 0.20 | (.31) |  | 0.34 | (.22) |  | -0.43 | (.36) |  | 0.28 | (.24) |
| High qualifications | 0.30 | (.31) |  | 0.50* | (.21) |  | -0.26 | (.36) |  | 0.75** | (.23) |
| Income (ref: low) |  |  |  |  |  |  |  |  |  |  |  |
| Medium | 0.07 | (.14) |  | -0.19 | (.17) |  | 0.20 | (.16) |  | 0.03 | (.18) |
| High | -0.14 | (.17) |  | -0.00 | (.19) |  | -0.05 | (.19) |  | 0.03 | (.20) |
| White ethnic group | -0.01 | (.25) |  | 0.13 | (.20) |  | -0.25 | (.30) |  | -0.26 | (.22) |
| Age | 0.64* | (.30) |  | -0.48 | (.32) |  | -0.95** | (.35) |  | -0.83* | (.35) |
| Female | 0.04 | (.12) |  | -0.15 | (.14) |  | 0.04 | (.12) |  | -0.20 | (.15) |
|  |  |  |  |  |  |  |  |  |  |  |  |
| Constant | 6.92** | (.55) |  | 5.98** | (.49) |  | 6.31** | (.64) |  | 5.39** | (.53) |
|  |  |  |  |  |  |  |  |  |  |  |  |
| Adjusted R^2^ | 0.32 | |  | 0.50 | |  | 0.36 | |  | 0.45 |  |
| Chi^2^ (20 *df*) | 26.44 | |  | 52.22 | |  | 30.77 | |  | 44.00 |  |
| Prob > F | 0.000 | |  | 0.00 | |  | 0.000 | |  | 0.000 |  |
| N | 1,069 | |  | 1,044 | |  | 1,067 | |  | 1,041 |  |
|  |  |  |  |  |  |  |  |  |  | |  |

^1^ Right party: Republicans (US), Conservative and Brexit parties (Britain). Other parties: all other candidates/parties.

** p≤0.01 *p≤0.05 (two-tailed test)

# APPENDIX 5: Full results of conjoint data models

|  |  |  |  | Scientists | | |  | Politicians | | |
| --- | --- | --- | --- | --- | --- | --- | --- | --- | --- | --- |
| Attribute |  | Levels |  | Marginal mean | s.e. | Sig |  | Marginal mean | s.e. | Sig |
|  |  |  |  |  |  |  |  |  |  |  |
| *Competence* |  | High quality work  Average quality work  Low quality work |  | 0.617  0.538  0.344 | (.01)  (.01)  (.01) | <0.05  <0.05  <0.05 |  | 0.578  0.519  0.404 | (.01)  (.01)  (.01) | <0.05  <0.05  <0.05 |
|  |  |  |  |  |  |  |  |  |  |  |
| *Transparency* |  | All data public  Some data public  No data public |  | 0.558  0.479  0.463 | (.01)  (.01)  (.01) | <0.05  <0.05  <0.05 |  | 0.569  0.473  0.458 | (.01)  (.01)  (.01) | <0.05  <0.05  <0.05 |
|  |  |  |  |  |  |  |  |  |  |  |
| *Representativeness* |  | In touch with people  A bit out of touch  Very out of touch |  | 0.578  0.485  0.436 | (.01)  (.01)  (.01) | <0.05  0.05  <0.05 |  | 0.589  0.487  0.423 | (.01)  (.01)  (.01) | <0.05  0.08  <0.05 |
|  |  |  |  |  |  |  |  |  |  |  |
| *Benevolence* |  | Very concerned about people  Somewhat concerned about people  Not very concerned about people |  | 0.558  0.510  0.432 | (.01)  (.01)  (.01) | <0.05  0.20  <0.05 |  | 0.584  0.500  0.416 | (.01)  (.01)  (.01) | <0.05  0.96  <0.05 |
|  |  |  |  |  |  |  |  |  |  |  |
| *Honesty* |  | Always admits mistakes  Sometimes admits mistakes  Rarely admits mistakes |  | 0.553  0.508  0.438 | (.01)  (.01)  (.01) | <0.05  0.28  <0.05 |  | 0.545  0.513  0.442 | (.01)  (.01)  (.01) | <0.05  0.09  <0.05 |
|  |  |  |  |  |  |  |  |  |  |  |
| *Communication* |  | Uses technical language  Presents in easy-to-understand way |  | 0.464  0.536 | (.01)  (.01) | <0.05  <0.05 |  | 0.465  0.535 | (.01)  (.01) | <0.05  <0.05 |
|  |  |  |  |  |  |  |  |  |  |  |
| *Independence* |  | Adjusts to politicians  Does not adjust to politicians |  | 0.395  0.605 | (.01)  (.01) | <0.05  <0.05 |  | 0.445  0.555 | (.01)  (.01) | <0.05  <0.05 |
|  |  |  |  |  |  |  |  |  |  |  |
| *Values* |  | Adjusts to business needs  Does not adjust to business needs |  | 0.500  0.501 | (.01)  (.01) | 0.94  0.94 |  | 0.545  0.455 | (.01)  (.01) | <0.05  <0.05 |
|  |  |  |  |  |  |  |  |  |  |  |

# APPENDIX 6: Full results of regression models of observational data – pooled country samples

|  | Trust in  scientists | |  | Trust in  politicians | |
| --- | --- | --- | --- | --- | --- |
| **Evaluations** |  |  |  |  |  |
| Scientists’ predictions often wrong | -1.86** | (.17) |  | -1.10** | (.19) |
| Gap on lockdowns-economy: scientists | 0.08 | (.29) |  | - | - |
| Gap on lockdowns-freedom: scientists | -0.25 | (.29) |  | - | - |
| Gap on lockdowns-economy: government | - | - |  | -1.13** | (.28) |
| Gap on lockdowns-freedom: government | - | - |  | -1.03** | (.28) |
| Scientists don’t follow Covid rules | -3.40** | (.23) |  | -0.70** | (.27) |
| Politicians don’t follow Covid rules | -0.10 | (.18) |  | -3.68** | (.21) |
|  |  |  |  |  |  |
| **Attitudes and values** |  |  |  |  |  |
| Partisanship^1^ (ref: Voted other party) |  |  |  |  |  |
| Voted right party | -0.31** | (.12) |  | -0.00 | (.13) |
| Did not vote | -0.42** | (.14) |  | -0.44** | (.16) |
| Authoritarian values | 0.11 | (.26) |  | 0.57 | (.30) |
| Religion (ref: None) |  |  |  |  |  |
| Christian | 0.14 | (.11) |  | 0.46** | (.13) |
| Other religion | 0.06 | (.18) |  | 0.21 | (.21) |
|  |  |  |  |  |  |
| **Science beliefs/fear** |  |  |  |  |  |
| High belief in science | 2.42** | (.23) |  | 1.79** | (.26) |
| Personal Covid fear | 0.48* | (.24) |  | 0.26 | (.28) |
| Communal Covid fear | 0.81** | (.29) |  | 1.15** | (.34) |
|  |  |  |  |  |  |
| **Demographics** |  |  |  |  |  |
| Education (ref: low qualifications) |  |  |  |  |  |
| Medium qualifications | 0.53** | (.17) |  | 0.27 | (,19) |
| High qualifications | 0.61** | (.16) |  | 0.54** | (.19) |
| Income (ref: low) |  |  |  |  |  |
| Medium | -0.03 | (.11) |  | 0.17 | (.13) |
| High | -0.09 | (.12) |  | 0.02 | (.14) |
| White ethnic group | 0.01 | (.15) |  | -0.46** | (.18) |
| Age | 0.01 | (.22) |  | -0.93** | (.25) |
| Female | -0.08 | (.09) |  | -0.19 | (.11) |
|  |  |  |  |  |  |
| Constant | 6.16** | (.35) |  | 5.43** | (.41) |
|  |  |  |  |  |  |
| Adjusted R^2^ | 0.42 | |  | 0.37 | |
| Chi^2^ (20 *df*) | 77.98 | |  | 61.70 | |
| Prob > F | 0.000 | |  | 0.000 | |
| N | 2,113 | |  | 2,108 | |

^1^ Right party: Republicans (US), Conservative and Brexit parties (Britain). Other parties: all other candidates/parties.

Contribution of each block of measures (R^2^) to predicting trust in scientists (‘Evaluations’: 0.36, ‘Attitudes and Values’: 0.09, ‘Science beliefs and fears’: 0.26, ‘Individual characteristics’: 0.09) and trust in politicians (‘Evaluations’: 0.31, ‘Attitudes and Values’: 0.06, ‘Science beliefs and fears’: 0.14, ‘Individual characteristics’: 0.03)

** p≤0.01 *p≤0.05 (two-tailed test)
